# Safety and pharmacodynamic effects of a pharmacological chaperone on α-galactosidase A activity and globotriaosylceramide clearance in Fabry disease: report from two phase 2 clinical studies

**DOI:** 10.1186/1750-1172-7-91

**Published:** 2012-11-24

**Authors:** Dominique P Germain, Roberto Giugliani, Derralynn A Hughes, Atul Mehta, Kathy Nicholls, Laura Barisoni, Charles J Jennette, Alexander Bragat, Jeff Castelli, Sheela Sitaraman, David J Lockhart, Pol F Boudes

**Affiliations:** 1 Division of Medical Genetics, Hôpital Raymond Poincaré (AP-HP), University of Versailles – St Quentin en Yvelines (UVSQ), Garches, 92380, France; 2 Medical Genetics Service, HCPA/UFRGS, Porto Alegre, Brazil; 3 Royal Free Campus, University College London, London, UK; 4 Royal Melbourne Hospital, Parkville, VIC, Australia; 5 New York University School of Medicine, New York, NY, USA; 6 University of North Carolina, Chapel Hill, NC, USA; 7 Amicus Therapeutics, Cranbury, NJ, USA

**Keywords:** Pharmacological chaperone, Conformational diseases, Protein-misfolding, Fabry disease, Lysosomal storage disorder

## Abstract

**Background:**

Fabry disease (FD) is a genetic disorder resulting from deficiency of the lysosomal enzyme α-galactosidase A (α-Gal A), which leads to globotriaosylceramide (GL-3) accumulation in multiple tissues. We report on the safety and pharmacodynamics of migalastat hydrochloride, an investigational pharmacological chaperone given orally at 150 mg every-other-day.

**Methods:**

Two open-label uncontrolled phase 2 studies of 12 and 24 weeks (NCT00283959 and NCT00283933) in 9 males with FD were combined. At multiple time points, α-Gal A activity and GL-3 levels were quantified in blood cells, kidney and skin. GL-3 levels were also evaluated through skin and renal histology.

**Results:**

Compared to baseline, increased α-Gal A activity of at least 50% was demonstrated in blood, skin and kidney in 6 of 9 patients. Patients’ increased α-Gal A activities paralleled the α-Gal A increases observed *in vitro* in HEK-293 cells transfected with the corresponding mutant form of the enzyme. The same 6 patients who demonstrated increases of α-Gal A activity also had GL-3 reduction in skin, urine and/or kidney, and had α-Gal A mutations that responded in transfected cells incubated with the drug. The 3 patients who did not show a consistent response *in vivo* had α-Gal A mutations that did not respond to migalastat HCl in transfected cells. Migalastat HCl was well tolerated.

**Conclusions:**

Migalastat HCl is a candidate pharmacological chaperone that provides a novel genotype-specific treatment for FD. It enhanced α-Gal A activity and resulted in GL-3 substrate decrease in patients with responsive *GLA* mutations. Phase 3 studies are ongoing.

**Trial registration:**

Clinicaltrial.gov: NCT00283959 and NCT00283933

## Background

Fabry disease (FD, OMIM 301500) is a rare, X-linked, multi-system genetic disorder [1]. Absent or deficient activity of lysosomal exoglycohydrolase α-galactosidase A (α-D-galactoside galactohydrolase, EC 3.2.1.22; α-Gal A) results in progressive accumulation of globotriaosylceramide (Gb_3_ or GL-3) and related glycosphingolipids within lysosomes in a variety of cell types, including capillary endothelial cells, and renal (podocytes, tubular cells, glomerular endothelial, mesangial and intersticial cells), and nerve cells [1]. The primary disease process starts in infancy. With age, progressive damage to vital organ systems develops leading to organ failure. End-stage renal disease and life-threatening cardiovascular or cerebrovascular complications limit life-expectancy [1]. Treatment with life-long enzyme replacement therapy (ERT) infusions is available [2,3]. However, due to concerns regarding convenience, cost and incomplete effects on disease progression, unmet medical needs remain and other treatments are under investigation [1,4].

Studies of α-Gal A indicate that mutant forms of the enzyme are often retained in the endoplasmic reticulum (ER) and prematurely degraded because of reduced stability or improperly folded conformations [5,6] as in other conformational or protein-misfolding diseases. This provides a rationale to use active site-specific pharmacological chaperones that bind and stabilize the nascent protein and restore efficient enzyme trafficking to lysosomes, the site of α-Gal A activity [5,7]. Migalastat HCl (AT-1001, GR181413A, 1-deoxygalactonojirimycin) is a low molecular weight iminosugar that is orally bioavailable and that acts as a pharmacological chaperone for α-Gal A, targetting α-Gal A mutants that maintain catalytic competence [5,8]. The mechanism of action of migalastat HCl is to bind and stabilize mutant α-Gal A initially in the ER, preventing misfolding and premature degradation and facilitating cellular trafficking to lysosomes where the breakdown of the GL-3 substrate can proceed [8,9].

We report on two phase 2 studies exploring the safety and pharmacodynamic responses to migalastat HCl in male patients with FD. In both studies, multiple parameters were evaluated to assess the effect of migalastat HCl on mutant α-Gal A activity and tissue GL-3.

## Patients and methods

Two open-label, uncontrolled, phase 2 studies (FAB-CL-202, NCT00283959 and FAB-CL-203, NCT00283933, respectively) were conducted to evaluate the safety, tolerability, and pharmacodynamics of migalastat HCl in males with FD. Alpha-galactosidase A activity and GL-3 levels were evaluated in blood, urine, skin and kidney. Patients were treated with migalastat HCl 150 mg orally every other day for 12 weeks (FAB-CL-202) or 24 weeks (FAB-CL-203). Both studies incorporated a treatment extension for a total duration of 48 weeks. The studies received Ethical Committee/Institutional Review Board (IRB) approval and were conducted according to accepted standards of Good Clinical Practice (ICH-GCP) and in agreement with the Declaration of Helsinki.

Safety and pharmacodynamic data are presented for the first 12 to 24 weeks of initial treatment and, when available, include data from week-48 renal biopsies.

### Patients

Inclusion and exclusion criteria were similar for both studies. After written informed consent, male patients between 18 and 65 years of age with a confirmed diagnosis of FD were enrolled (Table 1). A missense mutation in the *GLA* gene and residual α-Gal A activity of at least 3% of normal were required, as was the demonstration of an increase in α-Gal A activity in the presence of migalastat HCl in patient cultured lymphocytes. The initial criteria for enhancement required a relative increase in α-Gal A activity of at least 20% in the presence of 20 μM migalastat HCl. These criteria were later amended to a graded scale: if baseline activity was less than 1% of normal, it had to increase to at least 2% of normal; if activity was between 1% and 3% of normal, it had to at least double; if baseline activity was between 3% and 10%, it had to increase by at least 3% of normal; and if baseline activity was above 10%, it had to increase by at least 30%. Patients were to be naïve to ERT or willing to stop ERT for the duration of the study. Main exclusion criteria were significant disease or organ dysfunction, serum creatinine above 2 mg/dL and a QTc interval longer than 450 ms.

**Table 1:** Baseline characteristics

**Patient ID**	**Age (years)**	**eGFR (mL/min/1.73 m**^**2**^**)**	**24-hour protein (mg)**	**Left Ventricle Mass (g)**	***GLA *mutation**	**HEK assay: amenable mutation?**
2-0102	27	143.8	270	138	p.L415P	NO
2-0103	23	127.3	147	228	p.P259R	YES
2-0104	18	156.1	131	123	p.P259R	YES
2-0202	65	33	4640	312	p.R301Q	YES
3-0301	39	121.1	--^a^	225	p.F295C	YES ^b^
3-0302	31	134.5	--^a^	144	p.C94S	NO
3-0303	36	90.3	--^a^	175	p.R112C	NO
3-RF01	55	92.5	<100	231	p.N215S	YES
3-RFO3	47	135.7	150	200	p.P205T	YES

^a^ Value not available.

^b^ Mutation was amenable only when higher doses of migalastat hydrochloride were used.

The schedule of evaluation for α-Gal A activity and GL-3 parameters is shown in Table 2. Skin biopsies and kidney biopsies were obtained at multiple time points.

**Table 2:** Schedule of evaluations for α-Gal A activity (lymphocytes, peripheral blood mononuclear cells, skin and kidney) and GL-3 (plasma, 24-hour urine, skin and kidney)

	**Screening**	**Baseline**	**W4**	**W8**	**W12**	**W16**	**W20**	**W24**
**α-Gal A activity**								
Lymphocytes	+							
PBMCs	+	+	+	+	+	+^e^	+^e^	+^e^
Skin		+			+			+^e^
Kidney		+			+^d^			+^e^
**GL-3**								
Plasma	+	+	+	+	+	+^e^	+^e^	+^e^
Urine	+	+	+	+	+	+^e^	+^e^	+^e^
Skin								
LC/MS		+			+			+^e^
LM, EM^a^		+			+			+^e^
LM Semi Q^b^		+			+			+^e^
LM Quantitative^c^		+			+			+^e^
Kidney								
LC/MS		+			+^d^			+^e^
LM, EM^a^		+			+^d^			+^e^
LM Semi Q^b^		+			+^d^			+^e^
LM Quantitative^c^		+			+^d^			+^e^

W = week, α-Gal A = alpha-galactosidase A, PBMCs = Peripheral Blood Mononuclear Cells, LC/MS = Liquid Chromatography/Mass Spectrometry, GL-3 = globotriaosylceramide, LM = Light Microscopy, EM = Electron Microscopy, Q = Quantitative.

^a^ Multiple cell types using a subjective scoring scale of 0, 1, 2, or 3 based on increasing GL-3 content.

^b^ Peritubular capillaries using a modified Thurberg’s method ^14^.

^c^ Peritubular capillaries quantifying the number of GL-3 inclusion per cell using the the BLISS method ^15^.

^d^ FAB CL-202 (NCT00283959) only.

^e^ FAB CL-203 (NCT00283933) only.

### Measurement of α-Gal A activity

At screening, patient lymphocytes were isolated from blood and cultured in media containing interleukin-2 and phytohemagglutin. Lymphocyte α-Gal A activity was measured in cell lysates with a fluorimetric assay using 4-methylumbelliferyl α-D-galactopyranoside as a substrate in the presence of galactosamine. Alpha-Gal A activities were measured alone and after 3 days of *in vitro* incubation with 20 μM migalastat HCl [10]. Fulfillment of the enhancement criteria was reported as Yes/No.

At screening and multiple time points during studies, peripheral blood mononuclear cells (PBMCs) were isolated and α-Gal A activity was measured using the previously described method [10] (MDS Pharmaceutical Services, Lincoln, NE). Values were normalized to measured total protein using a colorimetric assay and α-Gal A activity was reported as nmol/hour/mg protein. Results were obtained as absolute values and as a percentage of normal. The normal value was determined to be 22.0 +/− 5.7 nmol/hr/mg protein (mean +/− SD measured in 16 healthy volunteers in the migalastat HCl phase 1 study FAB-CL-102).

At baseline and during treatment skin and kidney biopsies were analyzed for α-Gal A activity in aqueous homogenates using a non-GLP method derived from the PBMC method (MDS, Montreal, Canada). Timing of samples is reported in Table 2. Aqueous homogenization protocols for skin and kidney were developed and optimized for recovery of α-Gal A activity. Because enzyme activities may vary between tissues and even between cells within the same tissue, modifications were made to address technical issues associated with the limited amount of material and the potential for measured activities to be below the limit of detection in pre-treatment samples. Protocol modifications took into consideration the possibility for measured values to be above the level of detection but with insufficient sample volume to re-assay with dilution. The assay showed activity values varying by less than 10% in the kidney and by less than 20% in the skin. Control samples demonstrated good linearity over 50 serial 2-fold dilutions (average of <10% difference between predicted and measured signal). Measured values after 10-fold dilutions of the tissue samples were on average within 15% of predicted values for kidney, and within 25% of predicted values for skin.

### Measurement of α-Gal A activity in HEK-293 cells

In parallel with these studies, efforts to better define the range of α-Gal A mutant forms that are amenable to migalastat HCl were implemented. An *in vitro* α-Gal A gene transfection assay, specific for each individual mutation, was developed in HEK-293 cells [11]. Criteria for response in the presence of 10 μM migalastat HCl were both an absolute increase of 3% of normal activity and a 20% relative increase [11]. The HEK-293 assay was retrospectively used to define if a patient carrying a GLA mutation was amenable to migalastat HCl.

### Measurement of GL-3

#### Plasma GL-3

Plasma GL-3 levels were measured at MDS Pharma (Lincoln, NE). An aliquot of human plasma (with EDTA) containing the analyte and internal standard was extracted using a precipitation procedure under UV-shielded conditions. The extracted samples were analyzed by high performance liquid chromatography (HPLC) coupled with an AB/MDS Sciex API 4000 mass spectrometer (MS). Using a closely related assay in 205 healthy volunteers, the mean (SD) normal value was estimated to be 3.5 (1.3) μg/mL [12].

#### Urine GL-3 (u-GL-3)

Whole urine GL-3 measurements on 24-hour samples were performed using LC-MS/MS analysis. Initially, determination was done at MDS Pharma Services (lower limit of detection 1 μg/mL). As most samples had levels of GL-3 that were below this limit of quantification, a more sensitive analysis was used. Samples were analyzed at the Department of Genetic Medicine, Women’s and Children’s Hospital, North Adelaide, Australia. Urine sediment was subjected to sonication to fragment cells. Urinary lipids from sediment and supernatant were extracted by liquid-liquid extraction. Following chromatography, GL-3 isoforms were detected by electrospray ionization tandem mass spectrometry in multi-reaction mode. Total GL-3 was reported as the sum of five isoforms (C16:0, C20:0, C22:0, C24:0, C24:1). The lower limit of detection was approximately 1 ng/mL. To correct for urinary sediment cell content, GL-3 was normalized to total phosphatidylcholine (PC) determined in the same LC-MS/MS assay. Results were expressed in pmol GL-3/nmol PC [13]. Urine GL-3 was also measured in samples from healthy volunteers. The mean (SD) urine GL-3 value in 29 healthy male volunteers was 48.6 (13.0) pmol GL-3/nmol PC. Subject values above 74.6 pmol total GL-3/nmol PC (mean + 2 SDs) were considered abnormal.

#### Skin and kidney GL-3

GL-3 was measured in skin and kidney tissues with LC-MS/MS under non-GLP conditions on aqueous samples derived from tissue homogenates (MDS Pharma Services, Montreal, Canada). The homogenization protocol was the same as for α-Gal A activity. After chromatography, GL-3 isoforms were detected using electrospray ionization tandem mass spectrometry in multi-reaction mode. The amount of GL-3 in the sample was reported as the sum of nine isoforms. Results were expressed as micrograms GL-3/g of tissue. The analytical range of the assay was 0.1 to 5.0 μg/g of tissue.

#### Skin and kidney histology

GL-3 accumulation was measured by histology in skin and kidney biopsies in multiple cell types. Analysis was performed using both light microscopy (LM) on glutaraldehyde-fixed and plastic-embedded sections stained with methylene blue-Azure II, and by electron microscopy (EM). Pathologists were blinded to the timing of the sample.

In skin and kidney, a GL-3 score was assigned by a single pathologist based on a semi-quantitative scale, with zero representing no GL-3 and three representing the most severe GL-3 accumulation [14]_._ In kidney, cells included podocytes, distal tubular cells and peri-tubular capillary endothelial cells (PTCs) [14]. For PTCs, the cell type previously used to determine the primary measure of efficacy for registration studies [14], the sensitivity of GL-3 analysis was further improved by using a novel scoring system, the Barisoni Lipid Inclusion Scoring System (BLISS) [15]. Two pathologists independently counted the total number of GL-3 inclusions per capillary in all 50–180 recorded capillaries and summarized the average score per PTC. The final BLISS score was the average of both pathologists’ scores.

Distal kidney tubules were also qualitatively evaluated for GL-3. Two pathologists individually determined whether there were fewer, equal, or more GL-3 inclusions present at the available time points.

A qualitative analysis of glomerular sclerosis was performed. For each available time point, two pathologists independently determined the percentage of glomeruli that were normal or sclerotic and further sub-categorized them as globally or segmentally sclerotic.

Finally, pharmacodynamic responses for α-Gal A activity and GL-3 were summarized for each patient to determine an overall individual response (post-hoc analysis). A positive response for α-Gal A activity was defined as an increase of at least 50% above baseline. Changes in GL-3 parameters were assessed as a percentage decrease from baseline of ≥ 20% for u-GL-3 and ≥50% in GL-3 inclusion number in PTCs. Evidence of an overall response was considered to be present if enhancement of α-Gal A activity was associated with GL-3 reduction in skin and/or kidney.

### Safety

Adverse events (AE), serious adverse events (SAE), laboratory (hematology, chemistry, and urinalysis) and patient drops-outs were evaluated all along the studies and extension periods.

When appropriate, descriptive statistics (mean, SD) were provided. Because of the small number of patients and the uncontrolled nature of the studies, no inferential statistics were used. Results are presented as individual patient data.

## Results

Studies FAB CL-202 and FAB CL-203 recruited four and five subjects, respectively. The mean (SD) age at screening was 38 (15.4) years. The most frequent clinical manifestations at baseline were cold/heat intolerance (9/9), reduced sweating (9/9), angiokeratomas (8/9), acroparesthesias (7/9), hypoaccousia (5/9) and proteinuria (4/9). Three patients had hypertrophic cardiomyopathy. One patient had renal insufficiency (eGFR 33 mL/min/1.73 m^2^) (Table 1).

All patients carried a missense mutant α-Gal A that was considered to be responsive in the initial lymphocyte assay. When subsequently tested in the HEK-293 transfection assay, only six patients had mutations considered amenable to migalastat HCl (p.P205T, p.N215S, p.P259R two patients, p.F295C, p.R301Q) while three had mutations that did not show a sufficient enzymatic activity increase in HEK-293 cells incubated with the drug (p.C94S, p.R112C, p.L415P) (Table 1).

### Safety

Migalastat HCl was well tolerated. No subject discontinued treatment for an adverse event and there was no drop out for safety reasons. There were no deaths and no specific AE, serious adverse event (SAE), or laboratory abnormalities that were deemed related to migalastat HCl.

### α-Gal A activity

**Figure 1:**
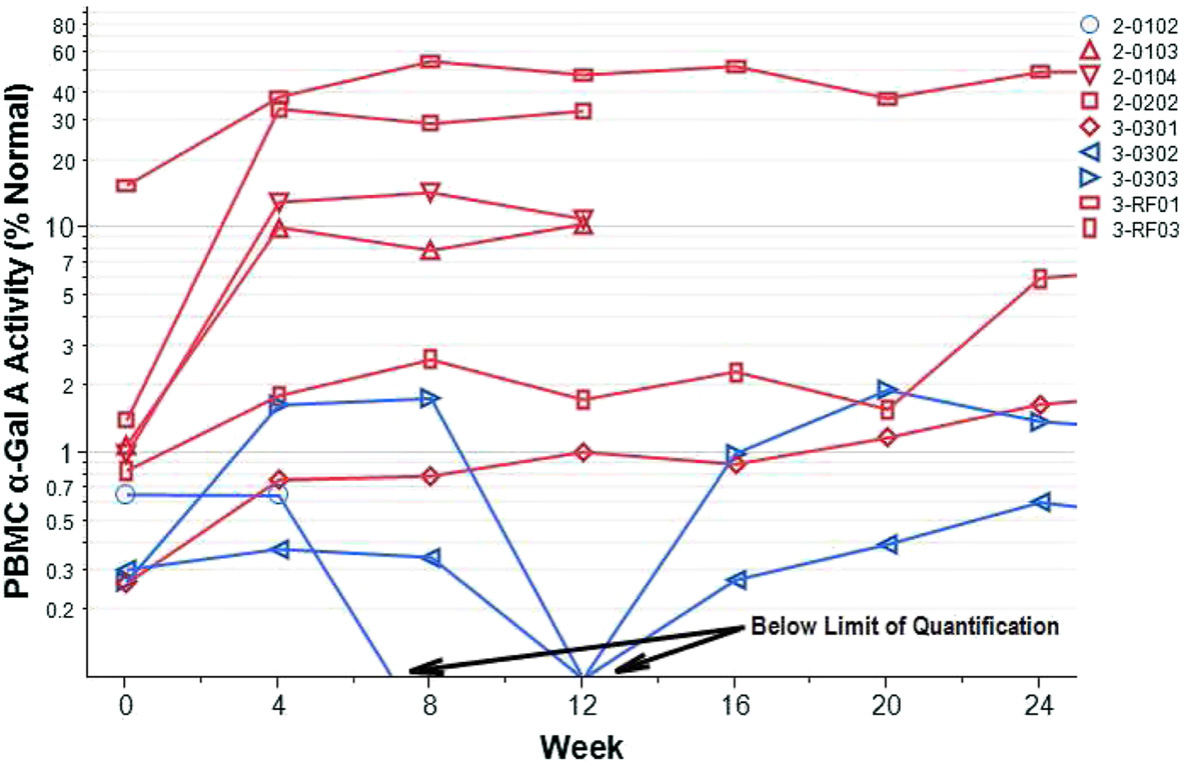
**PBMC α-Gal A activity (as a percentage of normal) by week of migalastat HCl treatment.** The red lines are for patients with mutations amenable to migalastat HCl as measured in the HEK-293 cell-based assay. The blue lines are for patients with non-responsive mutations. Values below 0.2 are below the limit of quantification (BLQ).

At baseline, PBMC α-Gal A activity ranged from 0.05 to 6.11 nmol/hour/mg protein (Table 3). The greatest activity corresponded to 16% of normal and in 8/9 subjects the activity was ≤ 1% of normal. During treatment, PBMC α-Gal A activity generally increased by the first time point and plateaued thereafter (Figure 1). For two subjects the maximum activity was seen at the last time point.

**Table 3:** α-Gal A activity in peripheral blood mononuclear cells (PBMCs, nmol/hr/mg protein)

	**Screening**	**Baseline**	**Week 4**	**Week 8**	**Week 12**	**Week 16**	**Week 20**	**Week 24**
FAB CL-202 (NCT00283959) ^1^								
2-0102^2^	0.15	0.14	0.14	0.18	0.12	-	-	-
2-0103	0.22	0.24	2.23	1.76	2.3	-	-	-
2-0104	0.5	0.21	2.88	3.18	3.36	-	-	-
2-0202	0.5	0.3	7.53	6.45	7.12	-	-	-
FAB CL-203 (NCT00283933) ^1^								
3-0301^2^	0.13	0.08	0.17	0.17	0.22	0.19	0.25	0.36
3-0302	0.18	0.10	0.08	0.07	BLQ	0.06	0.08	0.13
3-0303	BLQ	0.14	0.36	0.37	BLQ	0.22	0.42	0.3
3-RF01	6.11	2.6	8.46	12.1	10.6	11.5	8.35	10.9
3-RF03	0.04	0.2	0.3	0.57	0.38	0.51	0.34	1.32

^1^ Study number.

^2^ Patient ID.

BLQ = Below limit of quantification. Enzyme activity was reported as nmol/hour/mg protein.

In skin homogenates, baseline α-Gal A activity ranged from 0.2 to 37.3 nmol/hour/mg protein. Following treatment, increases in α-Gal A activity of at least 50% were noted for all subjects (Table 4).

**Table 4:** α-Gal A activity in skin and kidney (nmol 4-MU/hr/mg protein)

	**Skin**			**Kidney**		
	**Baseline**	**Week 12**	**Week 24**	**Baseline**	**Week 12**	**Week 24**
FAB CL-202 (NCT00283959) ^1^						
2-0102 ^2^	0.25	0.99	-	0.6	0.7	-
2-0103 ^2^	0.65	34.05	-	0.8	4.7	-
2-0104 ^2^	1.76	28.24	-	0.9	10.2	-
2-0202 ^2^	6.97	105	-	4.4	29.9	-
FAB CL-203 (NCT00283933) ^1^						
3-0301 ^2^	0.92	3.08	2.6	0.4	-	1.2
3-0302 ^2^	0.37	2.36	1.13	1.7	-	1.3
3-0303 ^2^	0.82	2.97	1.37	0.8	-	1.0
3-RF01 ^2^	37.29	139	133	21.6	-	157
3-RF03 ^2^	1.22	22.77	41.88	1.5	-	14.7

^1^ Study number.

^2^ Patient ID.

In kidney homogenates, α-Gal A activity at baseline ranged from 0.4 to 21.6 nmol/hour/mg protein. Following treatment, increases in α-Gal A activity of at least 50% were noted for 6/9 patients (Table 4).

### Plasma GL-3

At baseline, plasma GL-3 was between 1.12 and 4.82 μg/mL. No patient had values above normal. Week 12 data were not available for study FAB CL-202. One patient showed a transient increase of plasma GL-3, but otherwise there were no notable changes (data not shown).

### Urine GL-3

**Figure 2:**
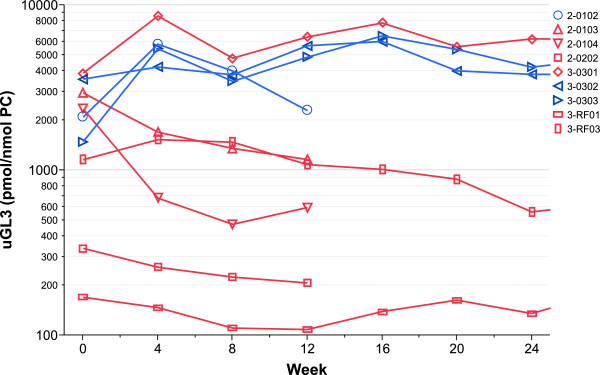
**Total urine GL-3 (in pmol/nmol phosphotidlycholine) by week of migalastat HCl treatment.** The red lines are for patients with an AT1001-amenable mutation in the HEK-293 cell-based assay, and the blue lines are for those with non-amenable mutations (Mutation p.F295C in patient 3–0301) was responsive only when high concentrations of migalastat HCl where used). Y axis: logarithmic scale.

All nine patients had elevated u-GL-3 prior to treatment; with a range from 170 to 3,876 pmol total GL-3/nmol PC. Compared to baseline, at the last time point decreased u-GL-3 levels of at least 20% were seen in 5/9 patients (Figure 2), all of whom had HEK-293 responsive mutations. Of the four patients without such a decrease, three had HEK-293 non-responsive mutations.

### Tissue GL-3 content

GL-3 in baseline renal biopsies ranged from 623 μg/g to 9,770 μg/g. During treatment GL-3 content decreased in five patients (4 of whom had HEK-293 responsive mutations) while 4 had an increase (Table 5). Of these four, two had HEK-293 responsive mutations.

For skin, the baseline GL-3 content ranged from 6.7 μg/g to 360 μg/g. During treatment GL-3 content decreased in 3 patients, all of whom had HEK-293 responsive mutations (Table 5).

**Table 5:** Skin and kidney GL-3 content at baseline, week 12 and week 24 (μg/g tissue)

	**Baseline**		**Week 12**		**Week 24**	
	**Skin**	**Kidney**	**Skin**	**Kidney**	**Skin**	**Kidney**
FAB CL-202 ^1^						
2-0102 ^2^	267	4170	269	3260	-	-
2-0103	337	9510	233	5590	-	-
2-0104	360	9770	309	9210	-	-
2-0202	10.3	4140	11.2	3250	-	-
FAB CL-203 ^1^						
3-0301^2^	223	3500	189	-	241	4960
3-0302	230	2100	259	-	349	2860
3-0303	246	623	344	-	300	2850
3-RF01	6.71	1340	9.38	-	10.3	4060
3-RF03	96.1	5300	64.8	-	41.5	3390

^1^ Study number.

^2^ Subject number.

### GL-3 kidney histology

As LM and EM results were generally consistent, only LM results are presented.

On the semi-quantitative LM scale, podocytes had consistently more GL-3 than any of the other 12 renal cell types evaluated. Even when other cells had minimal or no inclusions (i.e., score of 0), podocytes always contained GL-3 inclusions. At baseline 9/9 patients had a podocyte score of 3 (maximum). The second most affected cells were the collecting tubular cells. However these cells were not always present on slides, making evaluation problematic. At baseline, 2/2 patients’ collecting duct cells had a GL-3 inclusion score of 3.

For PTCs, the semi-quantitative and quantitative (BLISS) results were generally similar both at baseline and during treatment (data not shown). The quantitative method however was more sensitive as it could score inclusions even if the semi-quantitative score was “0”. The quantitative results are presented in Table 6. For 8/9 patients with available data, the baseline average count of GL-3 inclusions per cell ranged from 0.3 to 5.9. Between the first and last available biopsy the number of inclusions decreased in 4/8 patients, remained unchanged in 2/8, and increased in 2/8. Four of six patients who had HEK-293 responsive mutations had a score decrease, one had a score that did not change and one had a missing sample at baseline. Both patients who had an increase in score had mutations that were non-responsive in the HEK-293 assay. Of note, the inclusion count in PTCs did not always correlate with the changes in GL-3 on homogenized tissues.

**Table 6:** Quantitative GL3 scoring in kidney peri-tubular capillaries (PTC)

	**Visit**	**Reader 1**	**Reader 2**	**Average**
FAB CL-202^1^				
2-0102^2^	Baseline	2.3	2.3	2.3
	Week 12	5.7	6.6	6.2
2-0103	Baseline	4.4	7.4	5.9
	Week 12	0.3	0.3	0.3
2-0104	Baseline	2.3	3.5	2.9
	Week 48	0.9	1.4	1.2
2-0202	Baseline	0.5	0.2	0.3
	Week 12	0.8	0.3	0.5
	Week 48	0.3	0.3	0.3
FAB CL-203^1^				
3-0301^2^	Baseline	0.7	2.0	1.4
	Week 24	3.5	-	3.5
	Week 48	0	-	0
3-0302	Baseline	2.4	2.6	2.6
	Week 24	3.5	5.4	4.5
	Week 48	4.4	4.2	4.3
3-0303	Baseline	3.5	2.2	2.9
	Week 24	1.0	1.2	1.1
	Week 48	2.6	3	2.8
3-RF01^3^	Baseline	0.4	0.1	0.3
	Week 24	0.1	0.0	0.1
	Week 48	-	-	-
3-RF03^4^	Baseline	-	-	-
	Week 24	0.0	0.1	0.1
	Week 48	0.0	0.0	0.1

^1^ Study number.

^2^ Subject number.

^3^ Week 48 biopsy not evaluable.

^4^ Baseline biopsy not evaluable.

Average number of GL-3 inclusions per cell as measured in biopsy samples at different time points (BLISS).

Due to the variations in the number of glomeruli between biopsies, the assessment of glomerular sclerosis change was challenging (data not shown). At baseline, glomerulosclerosis was absent in 8/9 patients and absent during the study. For the remaining patient (2–02) only 2 non-sclerotic glomeruli were available at baseline. For this patient, glomerulosclerosis was noted at week 12 but was less marked at week 48.

### GL-3 skin histology

Only LM results are presented. Most patients had low scores (0 or 1) at baseline and GL-3 inclusions were mostly seen in capillaries (endothelial cells and pericytes), large vessels (endothelial cells and medial smooth muscle cells) or perineurium (data not shown).There was no correlation between the presence of angiokeratoma and GL-3 scores: two patients who had no GL-3 deposition had angiokeratomas. No skin cells were individually as severely and consistently affected as kidney podocytes and there was a large variability of cell scores between patients. At baseline, the only cells that had a score of 3 were the perineurium (4/7) and large vessel smooth muscle cells (2/7). Results were highly variable throughout the study (data not shown). For capillary endothelial cells, when comparison between baseline and week 12 and week 24 data was made, 2/9 had a decrease (both had mutations that were responsive in the HEK-293 assay), 3/9 an increase, 4/9 unchanged. The observed changes were all less than 1 scoring unit.

### Composite outcome

Table 7 provides a summary of α-Gal A and GL-3 responses to migalastat HCl. Six out of nine patients had responses in both α-Gal A and GL-3 parameters in skin and/or kidney, and all six had α-Gal A mutations that were responsive in the HEK-293 assay. Both patients with the same mutation (p.P259R) had consistent positive responses. The three subjects who did not demonstrate a clinical response had mutations that were not responsive in the HEK-293 assay.

**Table 7:** Summary of α-Gal A increase and GL-3 reduction for each patient and combined score according to HEK-293 response status

**Patient**	**2-0103**	**2-0104**	**2-0202**	**3-0301**	**3RF01**	**3RF03**	**2-0102**	**3-0302**	**3-0303**
Mutation	p.P259R	p.P259R	p.R301Q	p.F295C	p.N215S	p.P205T	p.L415P	p.C94S	p.R112C
HEK-293	R	R	R	R	R	R	NR	NR	NR
α-Gal A PBMC (at least +50%)	+	+	+	+	+	+	-	-	+
α-Gal A kidney (at least +50%)	+	+	+	+	+	+	-	-	-
α-Gal A skin (at least +50%)	+	+	+	+	+	+	+	+	+
u-GL3	+ (−60%)	+ (−75%)	+ (−38%)	-	+ (−20%)	+ (−52%)	-	-	-
GL-3 skin LC/MS	+ (−31%)	+ (−11%)	-	-	-	+ (−57%)	-	-	-
GL-3 kidney LC/MS	+ (−41%)	+ (−6%)	+ (−21%)	-	-	+ (−36%)	+ (−22%)	-	-
GL-3 kidney Inclusion (−50%)	+	+	-	+	+	^a^	-	-	-
Combined score	+	+	+	+	+	+	-	-	-

HEK-293 = transfection assay in Human Embryonic Kidney-293 cells, R = responsive mutation, NR = non-responsive mutation, α-Gal A = alpha galactosidase A, PBMCs = Peripheral Blood Mononuclear Cells, GL-3 = globotriaosylceramide, u = urine, s = skin, k = kidney, LC/MS = Liquid Chromatography - Mass Spectrometry.

^a^ baseline biopsy not evaluable.

## Discussion

Mutations in proteins that reduce stability or lead to protein misfolding are common causes of lysosomal storage diseases. Unstable or misfolded mutant enzymes are recognized by the cellular endoplasmic reticulum (ER) quality control system and prematurely degraded before reaching lysosomes. Some iminosugars have a high affinity for the active site of lysosomal enzymes, and can reversibly bind, stabilize and act as specific pharmacological chaperones [1,5,16,17]. Stabilized mutant can pass ER quality control more efficiently and traffic to lysosomes [7]. Once in lysosomes, the non-covalently bound chaperones can dissociate, freeing the enzyme to bind its substrate [5].

In FD, this approach was first demonstrated in patients’ lymphoblasts using 1-deoxygalactonojirimycin (migalastat HCl) [5]. Migalastat HCl mimics the terminal α-galactose of GL-3 and binds to the active site of α-Gal A with high affinity and specificity [6]. The binding increases the stability of the enzyme, shifts the folding in favor of the proper conformation and allows traffic to lysosomes [17].

The objectives of the current studies were to explore the safety and pharmacodynamics of migalastat HCl in 9 male FD patients given 150 mg orally every other day.

Results from a 12-week and a 24-week study with similar design were combined. There was a consistent increase in α-Gal A activity in PBMCs, skin and kidney in patients carrying responsive *GLA* mutations. In PBMCs, increases were rapid (week 4) and sustained over the duration of treatment. One patient, with a low baseline α-Gal A activity, showed a progressive increase with a maximum only reached at week 24. Some patients reached an enzyme activity in PBMCs of 30% to 50% of normal. Increase in α-Gal A activity were associated with substrate reduction, as demonstrated by a decrease in urinary GL-3 and GL-3 inclusions in renal PTCs.

These results indicate that the activity of mutated α-Gal A can be increased *in vivo* following administration of migalastat HCl. The binding of the drug to α-Gal A is reversible and is of lower affinity in the acidic environment of the lysosome. Furthermore, migalastat HCl is rapidly cleared from plasma (half-life 3–4 hours), whereas the lysosomal half-life of the enzyme is significantly longer (around 110–120 hours) [8]. This allows the enzyme to bind and turn over the GL-3 substrate without inhibition by the small molecule. The every-other-day regimen allows additional time for the chaperone to dissociate from the enzyme.

The *in vitro* HEK-293 cell-based assay [11] appears to predict the clinical pharmacodynamic response. Of note, both patients with the same p.P259R mutation showed similar pharmacodynamic response. As these studies included only 9 patients carrying 8 different missense mutations, results should be interpreted with caution. The predictive value of the assay will have to be confirmed in larger numbers of FD patients with additional mutations. This assay is currently used to select patients for phase 3 clinical studies.

FD is a rare, lifelong devastating disease, punctuated by acute complications that take many clinical forms [1]. FD phenotypic expression varies from one patient to the next, even within families harboring the same genotype. It is thus a challenge to select clinical outcome measures that can consistently demonstrate therapeutic efficacy [18]. Because GL-3 is the primary lysosomal substrate of α-Gal A, the deficient enzyme in FD, demonstrating a decrease in GL-3 could reflect treatment efficacy.

While GL-3 deposition in renal PTCs has been used as the primary outcome measure of efficacy for the approval of agalsidase beta [2], this choice may not be appropriate. Despite an overt clinical expression of the disease, our patients had limited amounts of GL-3 in capillary cells of the kidney and skin, and normal levels of GL-3 in plasma. Kidney histology revealed that GL-3 deposition was extensive in podocytes and collecting duct cells, but minimal in PTCs. A previously used histological method for quantifying GL-3 in PTCs [14] was not sensitive enough to evaluate the low levels of inclusions observed. Thus, a more sensitive method to quantify GL-3 PTC inclusions was developed [15]. Interestingly, the shedding into the urinary tract of tubular cells, and potentially podocytes, accounts for most of the urinary GL-3 in FD [19]_,_ especially when plasma GL-3 levels are low and glomerular filtration of GL-3 negligible. A decrease in u-GL-3 was observed in our subjects and has been advocated as a marker of efficacy in FD, however the clinical relevance remains controversial [13,20]. Some of the controversies stem from a lack of a reliable method for collection of samples and analysis [13,21]_._ To address these concerns, a new GLP assay for urine GL-3 was developed and validated, and will be used in ongoing migalastat HCl phase 3 studies.

In contrast to ERT, migalastat HCl is a small molecule that is excreted unchanged in the urine and can potentially reach podocytes. These cells are central to the renal pathophysiology of FD [22]. Migalastat HCl was generally well tolerated. However, these studies only enrolled 9 subjects and this should be interpreted with evident caution. Long-term extension data from phase 2 studies also indicate that the drug is well tolerated and, as of this writing, no severe adverse reactions related to treatment have been identified after four years of treatment.

A key issue for physicians treating FD patients is the selection of candidates for pharmacological chaperone therapy. Severe *GLA* defects that result in no residual α-Gal A activity are probably not addressable with chaperone therapy, and only patients who express missense mutants with low levels of activity were included. Different methods were explored to predict which patients would respond to migalastat HCl [8,11]. We initially recruited patients with missense mutations who had at least 3% of normal activity that increased by at least 20% when lymphocytes were incubated with migalastat HCl. However, the *ex vivo* lymphocyte assay is complex and cannot be readily performed under GLP conditions. Moreover, the lymphocyte assay did not always correlate with the *in vivo* PBMC assay. Ultimately, lymphocyte and PBMC assays are not useful in heterozygous FD females. In an X-linked disease, females are mosaics and isolated cells are a mix of cells with normal α-Gal A and ones with mutated α-Gal A. In females, an increase in activity with migalastat HCl might reflect the chaperoning of the wild-type enzyme. It has been increasingly recognized that females with FD can have significant clinical manifestations [23,24]. For these reasons, an *in vitro* assay was developed that could be used irrespective of sex [11].

In summary, migalastat HCl is a candidate pharmacological chaperone that provides a genotype-specific treatment for FD. When administered at an oral dose of 150 mg on an every-other-day regimen, it was well tolerated, increased α-Gal A activity in patients with responsive *GLA* mutations, and resulted in GL-3 substrate reduction. Phase 3 studies of migalastat HCl for FD are ongoing.

This study describes the first use in patients of an oral small molecule pharmacological chaperone, rather than using enzyme replacement therapy, to treat a lysosomal storage disorder. It shows for the first time in medicine that such a drug increases the activity, or effectively rescues the mutated and dysfunctional enzyme that patients with Fabry disease have expressed their entire lives. In addition to being a novel approach for the treatment of Fabry disease, stabilization of target proteins using small cell-permeable pharmacological chaperones, may represent a generally applicable rescue strategy for other diseases that result from improper protein folding and inefficient cellular targeting [25-27].

## Competing interests

DPG is an investigator for Amicus and has received research funding, consultancy fees, and travel expenses from Genzyme and Shire HGT. RG is a consultant and investigator for Actelion, Amicus, BioMarin, Genzyme and Shire HGT. DAH is a consultant for Amicus, Shire HGT, Genzyme, Actelion, has Speaker’s Bureau for Amicus, Shire HGT, Genzyme, and Actelion, and has received grants from Amicus, Shire HGT, and Genzyme. KN has received travel and research support and speaker's honoraria from Amicus, Shire HGT and Genzyme. AM has received honoraria, research funding, consultancy fees, and travel expenses from Shire HGT, Genzyme, Actelion, Protalix, and Amicus. AB, JC, SS, DJL and PFB are Amicus employees.

## Authors’ contributions

DPG, RG, DAH, AM and KN performed all clinical investigations. LB and CJJ completed the kidney biopsies reading. AB, JC, SS and DJL designed and performed experiments. DPG and PFB analyzed and interpreted the data, and wrote the manuscript. All the authors discussed the results and commented on the manuscript at all stages. All the authors have read and approved the final manuscript.

## References

[B1] Germain DP Fabry disease Orphanet J Rare Dis 2010 5 30 10.1186/1750-1172-5-30 21092187 PMC3009617

[B2] Eng CM Guffon N Wilcox WR Safety and efficacy of recombinant human a-galactosidase A – replacement therapy in Fabry's disease N Engl J Med 2001 345 9 16 10.1056/NEJM200107053450102 11439963

[B3] Schiffmann R Kopp JB Austin HA Enzyme replacement therapy in Fabry disease: a randomized controlled trial JAMA 2001 285 2743 2749 10.1001/jama.285.21.2743 11386930

[B4] Alfhadel M Sirrs S Enzyme replacement therapy for Fabry disease: some answers but more questions Ther Clin Risk Management 2011 7 69 82 10.2147/TCRM.S11987 PMC3061846 21445281

[B5] Fan JQ Ishii S Asano N Suzuki Y Accelerated transport and maturation of lysosomal a-galactosidase A in Fabry lymphoblasts by an enzyme inhibitor Nature Med 1999 5 112 115 10.1038/4801 9883849

[B6] Ishii S Chang HH Kawasaki K Mutant alpha-galactosidase A enzymes identified in Fabry disease patients with residual enzyme activity: biochemical characterization and restoration of normal intracellular processing by 1-deoxygalactonojirimycin Biochem J 2007 406 285 295 10.1042/BJ20070479 17555407 PMC1948963

[B7] Yam GH Zuber C Roth J A synthetic chaperone corrects the trafficking defect and disease phenotype in a protein misfolding disorder FASEB J 2005 19 12 18 10.1096/fj.04-2375com 15629890

[B8] Benjamin ER Flanagan JJ Schilling A The pharmacological chaperone 1-deoxygalactonojirimycin increases alpha-galactosidase A levels in Fabry patient cell lines J Inherit Metab Dis 2009 32 424 440 10.1007/s10545-009-1077-0 19387866

[B9] Khanna R Soska R Lun Y The pharmacological chaperone 1-deoxygalactonojirimycin reduces tissue globotriaosylceramide levels in a mouse model of Fabry disease Mol Ther 2010 18 23 33 10.1038/mt.2009.220 19773742 PMC2839206

[B10] Mayes JS Scheerer JB Sifers RN Donaldson ML Differential assay for lysosomal alpha-galactosidases in human tissues and its application to Fabry's disease Clin Chim Acta 1981 112 247 251 10.1016/0009-8981(81)90384-3 6263521

[B11] Wu X Katz E Della Valle C A pharmacogenetic approach to identify mutant forms of α-Galactosidase A that respond to a pharmacological chaperone for Fabry disease Hum Mutat 2011 2 965 977 21598360 10.1002/humu.21530 PMC3170878

[B12] Roddy TP Nelson BC Sung CC Liquid chromatography-tandem mass spectrometry quantification of globotriaosylceramide in plasma for long-term monitoring of Fabry patients treated with enzyme replacement therapy Clin Chem 2005 51 237 240 15514097 10.1373/clinchem.2004.038323

[B13] Fuller M Sharp PC Rozaklis T Urinary Lipid Profiling for the Identification of Fabry Hemizygotes and Heterozygotes Clin Chem 2005 51 688 694 10.1373/clinchem.2004.041418 15695328

[B14] Thurberg BL Rennke H Colvin RB Globotriaosylceramide accumulation in the Fabry kidney is cleared from multiple cell types after enzyme replacement therapy Kidney Int 2002 62 1933 1946 10.1046/j.1523-1755.2002.00675.x 12427118

[B15] Barisoni L Jennette JC Colvin R Novel Quantitative Virtual Microscopy-Based method to evaluate GL-3 inclusions in renal peritubular capillaries in patients with Fabry disease Arch Pathol Lab Med 2012 136 816 824 10.5858/arpa.2011-0350-OA 22742555

[B16] Fan JQ Ishii S Active-site-specific chaperone therapy for Fabry disease. Yin and Yang of enzyme inhibitors FEBS J 2007 274 4962 171 10.1111/j.1742-4658.2007.06041.x 17894781

[B17] Germain DP Fan JQ Pharmacological chaperone therapy by active-site-specific chaperones in Fabry disease: in vitro and preclinical studies Int J Clin Pharmacol Ther 2009 47 S111 7 20040321

[B18] Banikazemi M Bultas J Waldek S Agalsidase-beta therapy for advanced Fabry disease: a randomized trial Ann Intern Med 2007 146 77 86 17179052 10.7326/0003-4819-146-2-200701160-00148

[B19] Kitagawa T Ishige N Suzuki K Non-invasive screening method for Fabry disease by measuring globotriaosylceramide in whole urine samples using tandem mass spectrometry Mol Genet Metab 2005 85 196 202 10.1016/j.ymgme.2005.01.007 15979031

[B20] Schiffmann R Waldek S Benigni A Auray-Blais C Biomarkers of Fabry Disease Nephropathy Clin J Am Soc Nephrol 2010 5 360 364 10.2215/CJN.06090809 19965549

[B21] Auray-Blais C Millington DS Barr C Young SP Mills K Clarke JT Gb(3)/creatinine biomarkers for Fabry disease: issues to consider Mol Genet Metab 2009 97 237 10.1016/j.ymgme.2009.04.006 19428279

[B22] Najafian B Svarstad E Bostad L Progressive podocyte injury and globotriaosylceramide (GL-3) accumulation in young patients with Fabry disease Kidney Int 2011 79 663 670 10.1038/ki.2010.484 21160462 PMC3640823

[B23] Whybra C Kampmann C Willers I Anderson-Fabry disease: clinical manifestations of disease in female heterozygotes J Inherit Metab Dis 2001 24 715 724 10.1023/A:1012993305223 11804208

[B24] Wilcox WR Oliveira JP Hopkin RJ Females with Fabry disease frequently have major organ involvement: lessons from the Fabry Registry Mol Genet Metab 2008 93 112 128 10.1016/j.ymgme.2007.09.013 18037317

[B25] Kuznetsov G Nigam SK Folding of secretory and membrane proteins. N Engl J Med 1998 339 1688 1695 10.1056/NEJM199812033392307 9834307

[B26] Bradbury J Chaperones: Keeping a close eye on protein folding Lancet 2003 361 1194 95 10.1016/S0140-6736(03)12975-3 12686051

[B27] Cohen FE Kelly JW Therapeutic approaches to protein-misfolding diseases Nature 2003 426 905 909 10.1038/nature02265 14685252

